# Foliar Supplementation with NPK Stimulates Carbon Metabolism and Improves Water Use Efficiency and Off-Season Maize Crop Yield

**DOI:** 10.3390/plants15213484

**Published:** 2026-11-08

**Authors:** Leila Bernart Vilela, Sirlene Lopes Oliveira, Amanda Ferraresi Roberto, Laura Tarrafel da Silva, Amanda Ribeiro Peres Portugal, Letusa Momesso, Carlos Alexandre Costa Crusciol

**Affiliations:** 1 Department of Agronomy, Center for Agricultural Sciences, State University of Londrina (UEL), Londrina 86057-970, PR, Brazil; leilabernart@uel.br; 2 Federal Institute of Education, Science and Technology of Mato Grosso (IFMT), Sorriso 78890-000, MT, Brazil; sirlene.oliveira@ifmt.edu.br; 3 Department of Crop Science, College of Agricultural Sciences, São Paulo State University (UNESP), Botucatu 18610-034, SP, Brazil; amanda.ferraresi@unesp.br (A.F.R.); laura.tarrafel@unesp.br (L.T.d.S.); amandarperesagro@gmail.com (A.R.P.P.); 4 Department of Agriculture, School of Agriculture, Federal University of Goiás (UFG), Goiânia 74690-900, GO, Brazil; letusamomesso@gmail.com

**Keywords:** foliar NPK fertilization, nutritional status, carbon assimilation, carbohydrate metabolism, off-season cropping

## Abstract

Maize production in Brazil is concentrated in the off-season, after soybean harvest. Reduced water availability can compromise maize growth and productivity. Foliar fertilization may improve crop development and yield, although evidence on simultaneous application of macronutrients remains limited. This study evaluated the effects of foliar nitrogen–phosphorus–potassium (NPK) formulations application on off-season maize physiology and grain yield. We compared foliar NPK application with an untreated control over two growing seasons. Leaf nutritional status, photosynthetic enzymes (ribulose-1,5-bisphosphate carboxylase/oxygenase [Rubisco] and phosphoenolpyruvate carboxylase [PEPcase]), gas exchange, oxidative stress indicators (H_2_O_2_ and MDA), antioxidant enzymes (superoxide dismutase [SOD] and catalase [CAT]), protein and sugar concentrations, yield components, and grain yield were evaluated. Foliar NPK application improved maize nutritional status and photosynthetic performance Rubisco activity, gas exchange, water use efficiency (WUE) in 2020, and protein and sugar concentrations. Foliar NPK reduced hydrogen peroxide (H_2_O_2_) accumulation and lipid peroxidation, measured as malondialdehyde (MDA) in both seasons, accompanied by lower antioxidant enzyme activities, suggesting reduced oxidative pressure and antioxidant demand. Grain yield increased by 0.72 Mg ha^−1^ (11.5%) and 0.64 Mg ha^−1^ (12.0%) in the first and second seasons, respectively, compared with untreated maize. NPK improved physiological performance and grain yield of off-season maize, supporting its use as a complementary practice.

## 1. Introduction

Maize is a globally important crop, as it is one of cereals that can be cultivated under different soil conditions and environments [1]. The cultivation of off-season maize (after the summer soybean harvest) has expanded in recent years and currently accounts for approximately 80% of total Brazilian maize production [2,3]. However, off-season maize cultivation is highly dependent on climatic conditions and is particularly vulnerable to variability in water availability [4]. In this context, the irregularity of rainfall, characterized by reduced precipitation and uneven distribution after sowing, intensifies water stress, often associated with high temperatures [5]. During the reproductive stages, water deficit and elevated temperatures significantly impair pollination and grain filling, resulting in substantial yield losses [6].

Reproductive organs (tassels, silks, and developing kernels) are particularly sensitive to heat and drought, which can lead to pollination failure and increased kernel abortion [7,8,9,10]. Maize yield reductions of approximately 40% have been reported for water deficits during critical stages such as flowering and grain filling [7,11]. Temperatures above 30–35 °C during these stages also reduce pollen viability, kernel number, and kernel weight while accelerating crop development and shortening the grain-filling period [7,8,9]. When drought and heat occur simultaneously, yield losses exceed those observed under single stress conditions, frequently reaching 30–60% or more [12,13,14,15,16]. The physiological effects of combined heat and drought stress are attributable to the accumulation of reactive oxygen species (ROS), which negatively affect photosynthesis, stomatal conductance, and grain filling and accelerate leaf senescence [13,14,15]. Management strategies capable of mitigating stress-induced limitations on plant development are essential to ensure maize productivity under unfavorable environmental conditions.

Foliar fertilization has been widely explored as a complementary approach to soil fertilization due to its ability to provide rapid nutrient uptake, contributing to the maintenance of vegetative growth and supporting plant physiological performance, particularly under adverse conditions [17,18,19,20]. Additionally, foliar fertilization can reduce abiotic stress in crops by protecting the photosynthetic apparatus, activating the antioxidant defense system, improving water use efficiency, and regulating stomata [20,21,22,23,24].

Fertilization with nitrogen (N), phosphorus (P), and potassium (K) is a key determinant of vegetative growth and maize productivity, promoting increases in plant height, leaf area, dry matter accumulation, and grain yield [25,26]. N is essential for the synthesis of proteins, chlorophylls, cytochromes, DNA, and enzymes [27], whereas P is involved in root formation and energetic processes, such as photosynthesis and cellular respiration [28]. K, in turn, regulates plant water balance and stomatal opening, being essential for enzyme activation and protein synthesis [27].

Previous studies have demonstrated that foliar application of N, P, and K is associated with stress mitigation and enhanced photosynthetic activity [23,29,30]. Foliar N application has been shown to enhance antioxidant activity, improved net photosynthesis and ribulose-1,5-bisphosphate carboxylase/oxygenase (Rubisco) activity, ultimately leading to increases in yield components and grain yield [31]. Similarly, foliar P application has been associated with increased photosynthetic activity, antioxidant activity, and crop yield [20,23]. One of these studies demonstrated that the nitrogen present in the phosphorus source [Monoammonium phosphate (MAP)] contributed significantly to improvements in physiological performance and crop productivity, indicating that the combined application of these nutrients may be more effective than their individual application [32]. Foliar K application has also been investigated, with results demonstrating reduced oxidative damage, improved ionic balance, enhanced photosynthetic performance, and increased grain yield [33].

Although nitrogen–phosphorus–potassium (NPK) formulations are widely used for soil fertilization, the physiological responses to their combined foliar application have received comparatively less attention [34]. Nutrient homeostasis depends on complex physiological and metabolic interactions among nutrients, which collectively influence plant growth and function [35]. In this context, combined foliar NPK application may represent a complementary nutritional strategy capable of influencing photosynthetic metabolism and physiological performance in plants receiving conventional soil fertilization [36,37]. Previous research has reported improvements in crop growth, nutrient uptake, and grain yield following foliar application of combined NPK formulations, including nano-NPK in maize [38]. Nevertheless, the extent to which combined foliar NPK application affects carbon assimilation, antioxidant metabolism, and their relationships with grain yield remains insufficiently characterized in off-season maize grown under tropical field conditions. This knowledge gap is particularly relevant in production environments exposed to climatic variability, where periods of water limitation and high temperatures may restrict nutrient acquisition and impair plant physiological performance.

In this context, the present study aimed to investigate the influence of foliar application of NPK on physiological and biochemical processes, production components, and grain yield of off-season maize. We hypothesized that the combined foliar application of NPK enhances photosynthetic metabolism and water-use efficiency, thereby improving plant performance and grain yield of off-season maize grown under tropical field conditions.

## 2. Results

The *p*-values for the effects of foliar nitrogen, phosphorus, and potassium (NPK) application on leaf nutrient concentrations, gas exchange, photosynthetic enzyme activities, oxidative stress indicators, antioxidant enzyme activities, protein and soluble sugar concentrations, yield components, and grain yield during the 2019 and 2020 growing seasons are provided in the Supplementary Material (Table S3).

### 2.1. Climate Characteristics

**Figure 1:**
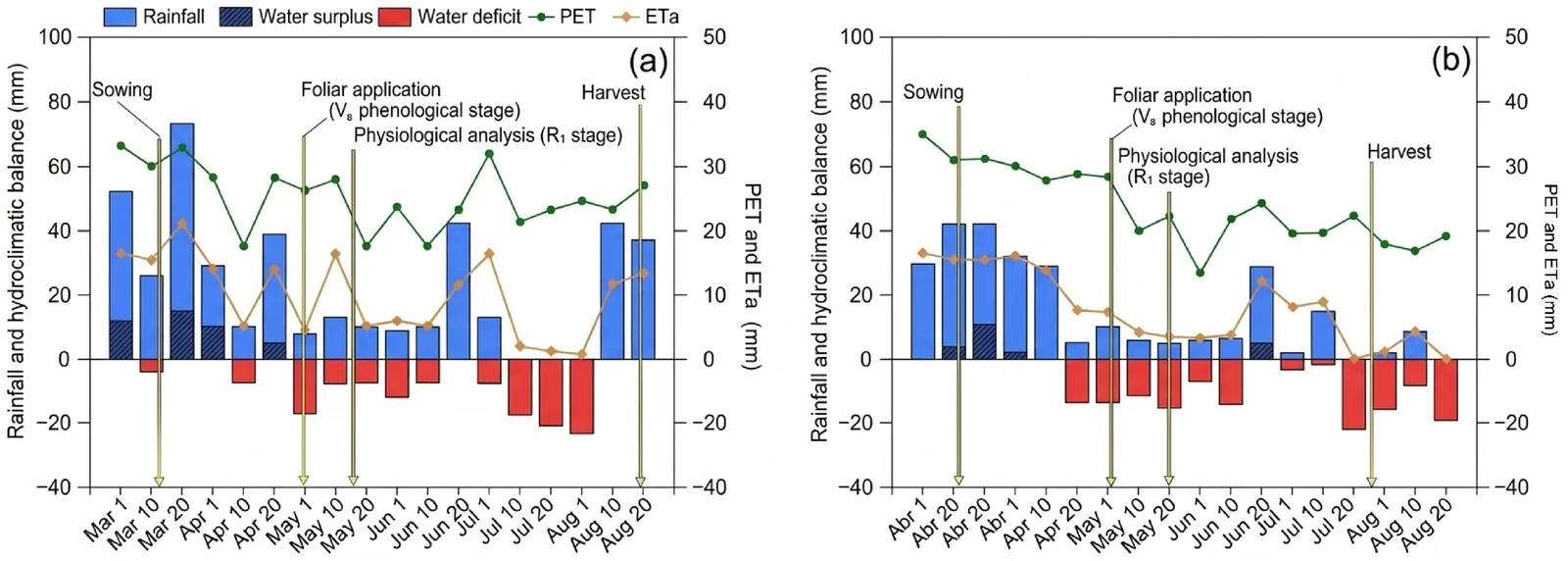
Hydroclimatic balance during the growth of off-season maize ((**a**), 2019; (**b**), 2020). PET: Potential evapotranspiration; ETa: actual evapotranspiration. The arrows indicate the timing of spraying and sampling.

Figure 1 shows the climatic water balance during the two off-season maize growing seasons (2019 and 2020), with rainfall distribution, water surplus/deficit, and potential evapotranspiration (PET) and actual evapotranspiration (ETa) dynamics.

The two maize growing seasons were characterized by limited water availability throughout most of the crop cycle, except during the early stages of vegetative development (Figure 1). Total precipitation was 319 and 293 mm in 2019 and 2020, respectively. Although precipitation was slightly higher in 2019, rainfall was unevenly distributed in both growing seasons, with water deficits becoming more pronounced as crop development progressed. In 2020, rainfall was concentrated during the early vegetative stages, followed by more frequent and persistent water deficits from May onward. Foliar NPK application at the V_8_ stage occurred near the transition from relatively favorable water availability to increasing water limitation, particularly in 2020.

### 2.2. Plant Nutrition

**Figure 2:**
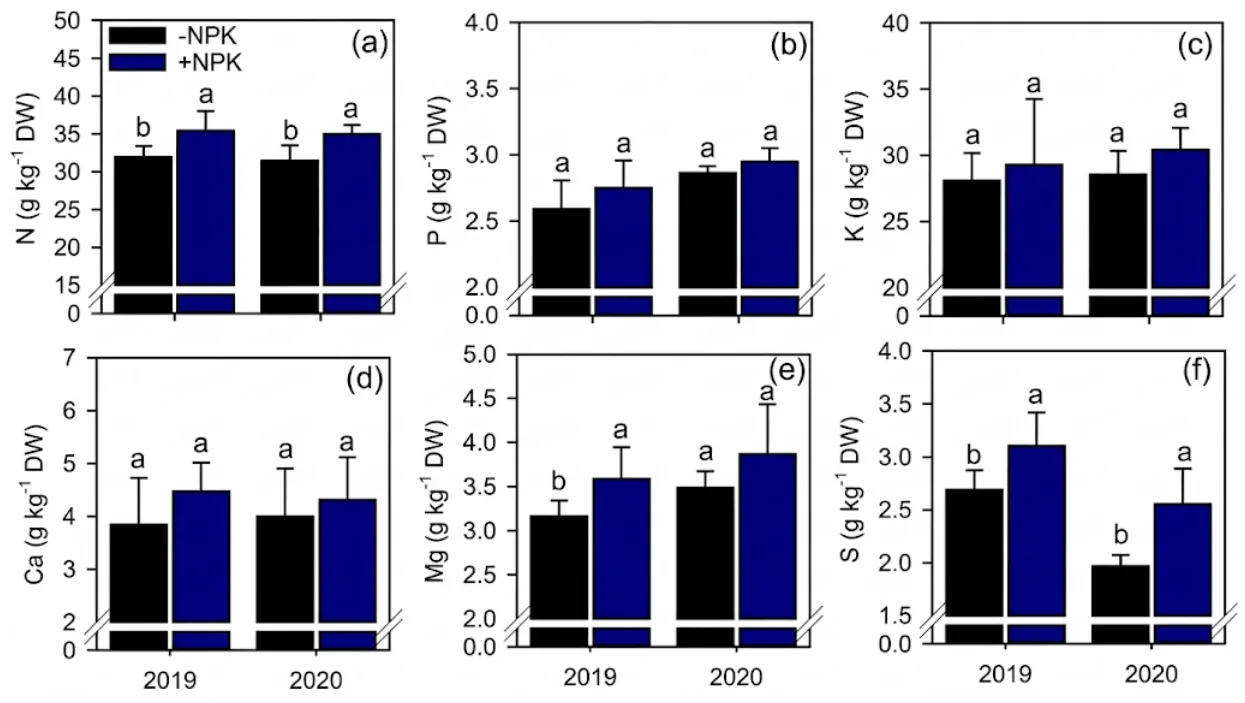
Maize leaf (**a**) nitrogen (N); (**b**) phosphorus (P); (**c**) potassium (K); (**d**) calcium (Ca); (**e**) magnesium (Mg); and (**f**) sulfur (S) concentrations as a function of the presence or absence of foliar NPK application (+NPK or −NPK). Different letters indicate significant differences between treatments in the same year according to the F-test (*p* ≤ 0.10). Error bars indicate the standard deviation (*n* = 12).

Foliar NPK application significantly increased maize leaf nitrogen (N) concentrations by 10.7% and 11.4% in the first and second growing seasons, respectively (Figure 2a). Leaf magnesium (Mg) concentrations were also significantly increased by 13.3% in the first growing season (Figure 2e). Leaf phosphorus (P), potassium (K), and calcium (Ca) concentrations did not differ significantly between treatments (Figure 2b–d). Leaf sulfur (S) concentrations increased by 15.2% and 29.8% in the first and second growing seasons, respectively (Figure 2f).

**Figure 3:**
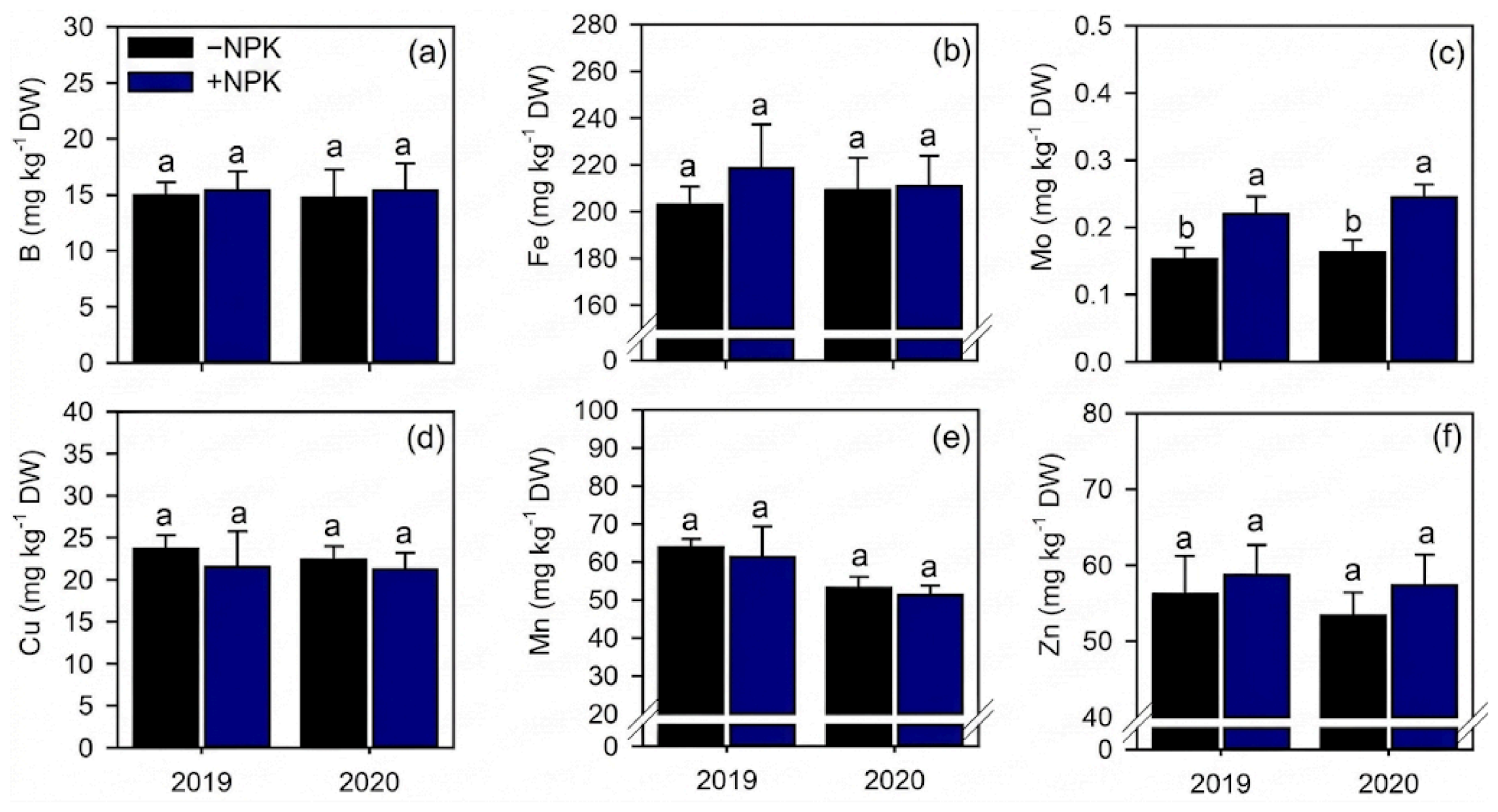
Maize leaf (**a**) boron (B); (**b**) iron (Fe); (**c**) molybdenum (Mo); (**d**) copper (Cu); (**e**) manganese (Mn); and (**f**) zinc (Zn) contents as a function of the presence or absence of foliar NPK application (+NPK or −NPK). Different letters indicate significant differences between treatments in the same year according to the F-test (*p* ≤ 0.10). Error bars indicate the standard deviation (*n* = 12).

Leaf micronutrient concentrations were not significantly different between +NPK and −NPK in 2019 and 2020, with the exception of molybdenum (Mo) (Figure 3). Foliar NPK application increased leaf Mo content significantly in both years, with increases of 44.3% in 2019 and 50.8% in 2020 compared to −NPK.

### 2.3. Photosynthetic Parameters and Carbon Assimilation

**Figure 4:**
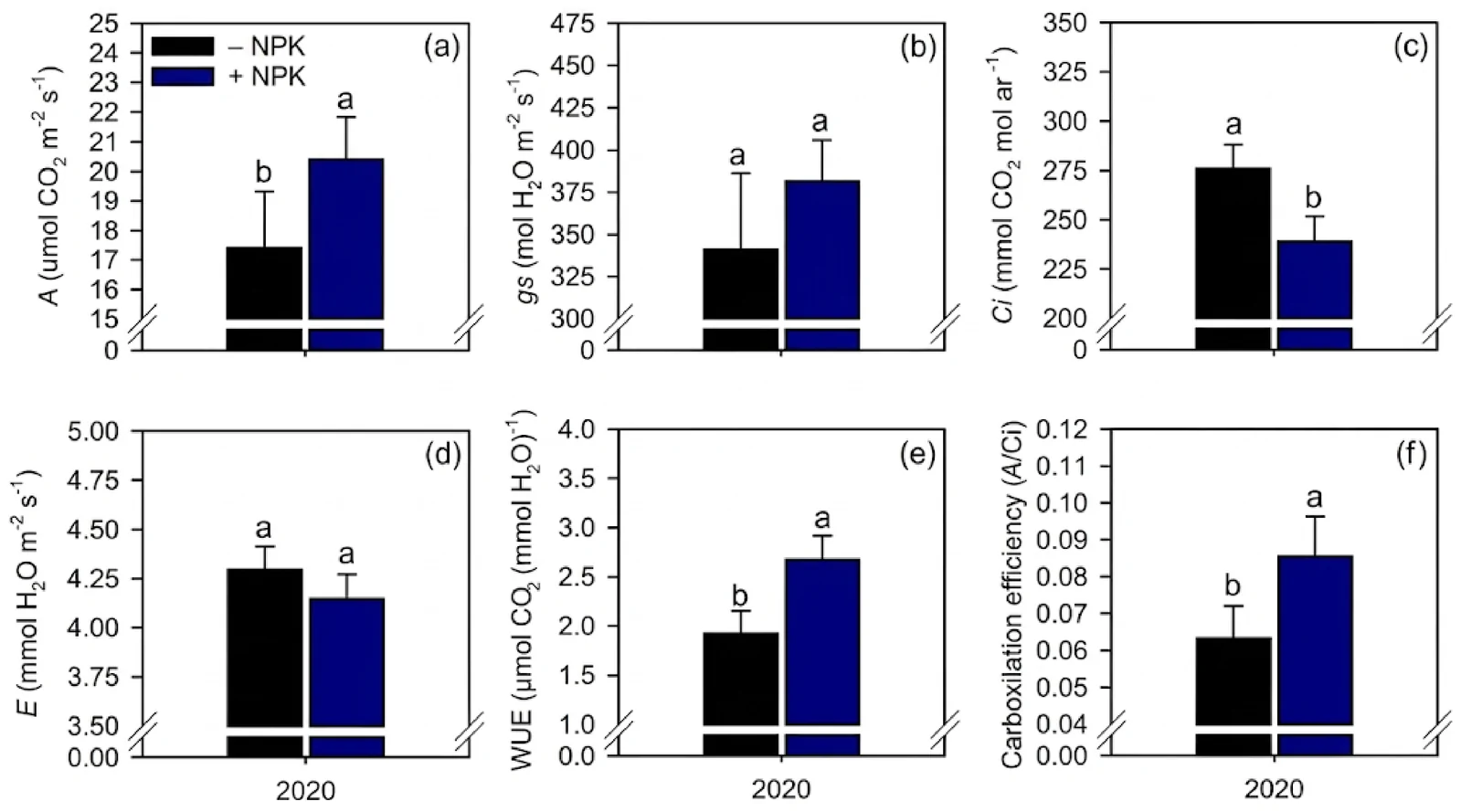
Off-season maize (**a**) net photosynthetic rate (*A*); (**b**) stomatal conductance (*gs*); (**c**) intercellular CO_2_ concentration (*Ci*); (**d**) foliar transpiration (*E*); (**e**) water use efficiency (WUE); and (**f**) carboxylation efficiency (*A*/*Ci*) as a function of the presence or absence of foliar NPK application (+NPK or −NPK) in the 2020 growing season. Different letters indicate significant differences between treatments according to the F-test (*p* ≤ 0.10). Error bars indicate the standard deviation (*n* = 12).

Foliar NPK application significantly improved the photosynthetic activity of off-season maize in 2020. Compared to −NPK, +NPK increased net photosynthetic rate (*A*) by 26.3% (Figure 4a). Due to the improvement in *A*, water use efficiency (WUE) and carboxylation efficiency (*A*/*Ci*) increased by 42.5% and 50%, respectively (Figure 4e,f). By contrast, +NPK decreased intercellular CO_2_ concentration (*Ci*) 13.4% compared to −NPK (Figure 4c,d).

**Figure 5:**
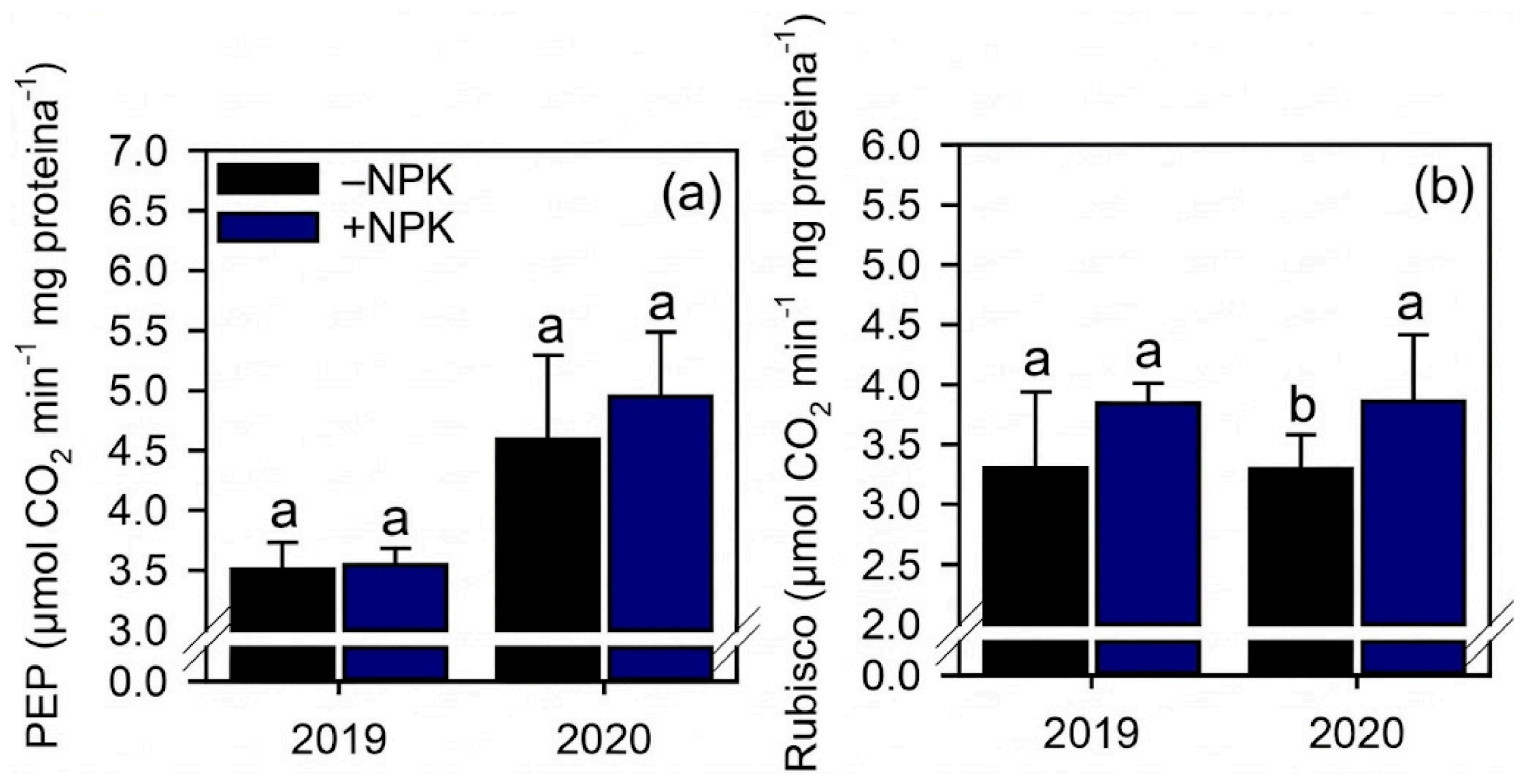
Maize leaf activities of the enzymes (**a**) PEP carboxylase and (**b**) Rubisco as a function of the presence or absence of foliar NPK application (+NPK or −NPK). Different letters indicate significant differences between treatments in the same year according to the F-test (*p* ≤ 0.10). Error bars indicate the standard deviation (*n* = 12).

Foliar NPK application resulted in distinct responses in the activities of the carbon-assimilation enzymes phosphoenolpyruvate carboxylase (PEPcase) and ribulose-1,5-bisphosphate carboxylase/oxygenase (Rubisco) (Figure 5). PEPcase activity remained relatively unchanged between +NPK and −NPK treatments in both growing seasons, showing no significant response to foliar nutrient supplementation. In contrast, a significant increase in Rubisco activity was detected (+17%) only in 2020 with the application of NPK. Thus, while PEPcase activity remained unaffected by foliar NPK application, Rubisco exhibited a more pronounced response, particularly in the second growing season.

### 2.4. Antioxidant Activity

**Figure 6:**
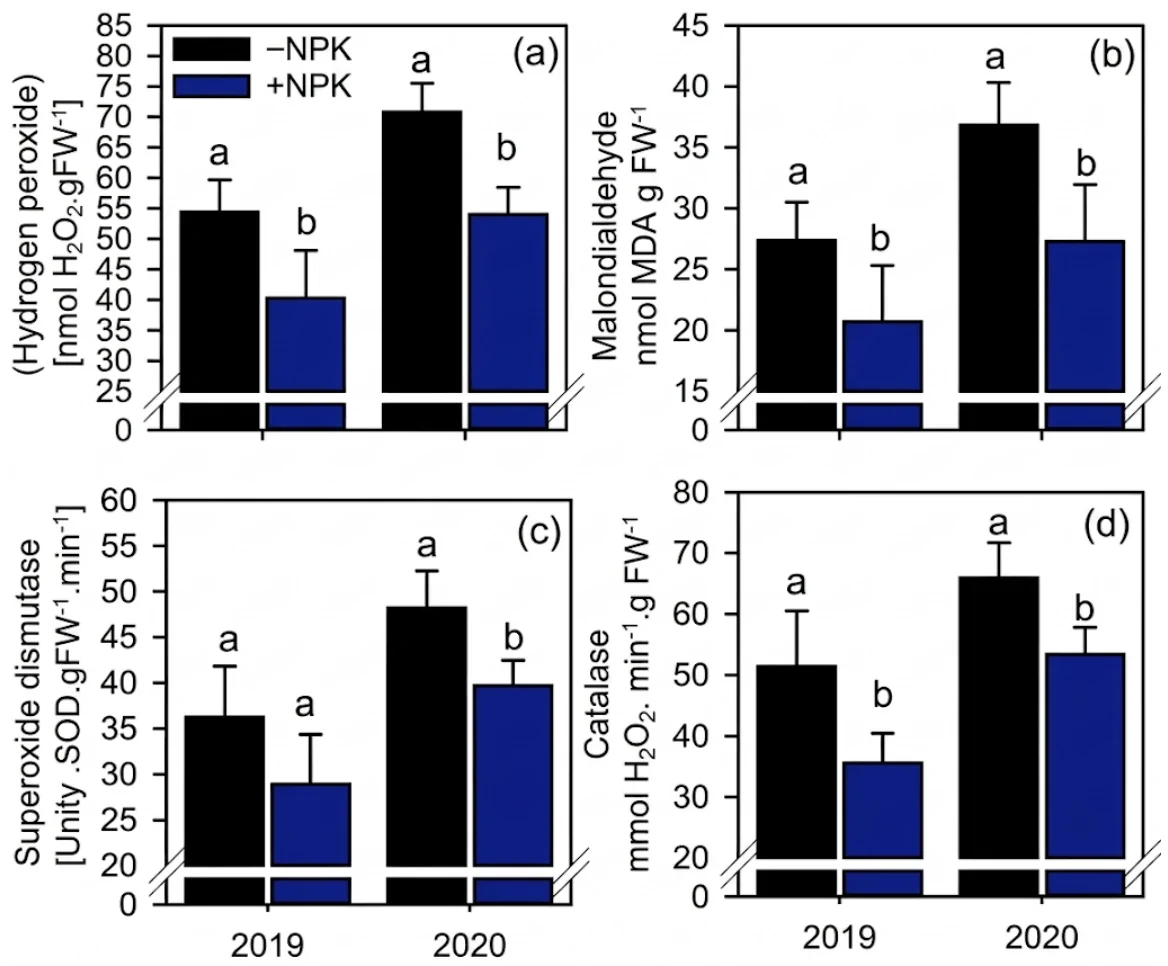
Off-season maize (**a**) hydrogen peroxide (H_2_O_2_); (**b**) malondialdehyde (MDA); (**c**) superoxide dismutase (SOD); (**d**) Catalase (CAT) as a function of the presence or absence of foliar NPK application (+NPK or −NPK) in the 2019 and 2020 growing seasons. Different letters indicate significant differences between treatments according to the F-test (*p* ≤ 0.10).

Plants that did not receive foliar NPK application exhibited higher levels of oxidative stress, as indicated by hydrogen peroxide (H_2_O_2_) and malondialdehyde (MDA) concentrations (Figure 6). Conversely, foliar NPK application reduced the levels of these compounds by approximately 25% in both growing seasons. In addition to the reduction in oxidative stress markers, the activities of the antioxidant enzymes superoxide dismutase (SOD: 18% in 2020) and catalase (CAT: 31% and 19%, in 2019 and 2020, respectively) also decreased in both growing seasons.

### 2.5. Protein and Total Soluble Sugar Content

**Figure 7:**
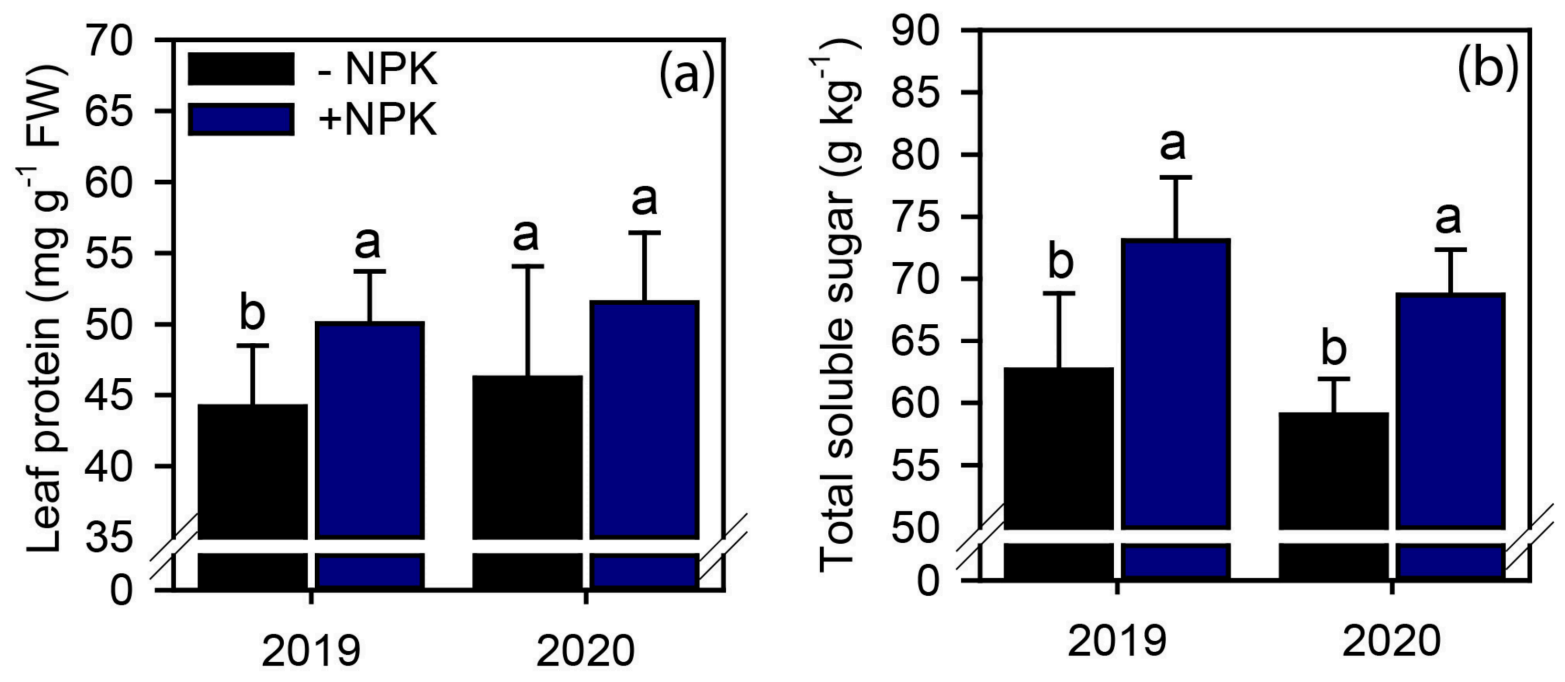
Off-season maize leaf (**a**) protein concentration and (**b**) total soluble sugar concentration as a function of the presence or absence of foliar NPK application (+NPK or −NPK). Different letters indicate significant differences between treatments according to the F-test (*p* ≤ 0.10).

Foliar NPK application significantly increased maize leaf protein and total soluble sugar content. Compared with −NPK, +NPK increased protein content by 13.3% only in 2019 (Figure 7a) and total soluble sugar content by 16.7% and 16.3% in 2019 and 2020, respectively (Figure 7b).

### 2.6. Agronomic Parameters and Grain Yield

**Figure 8:**
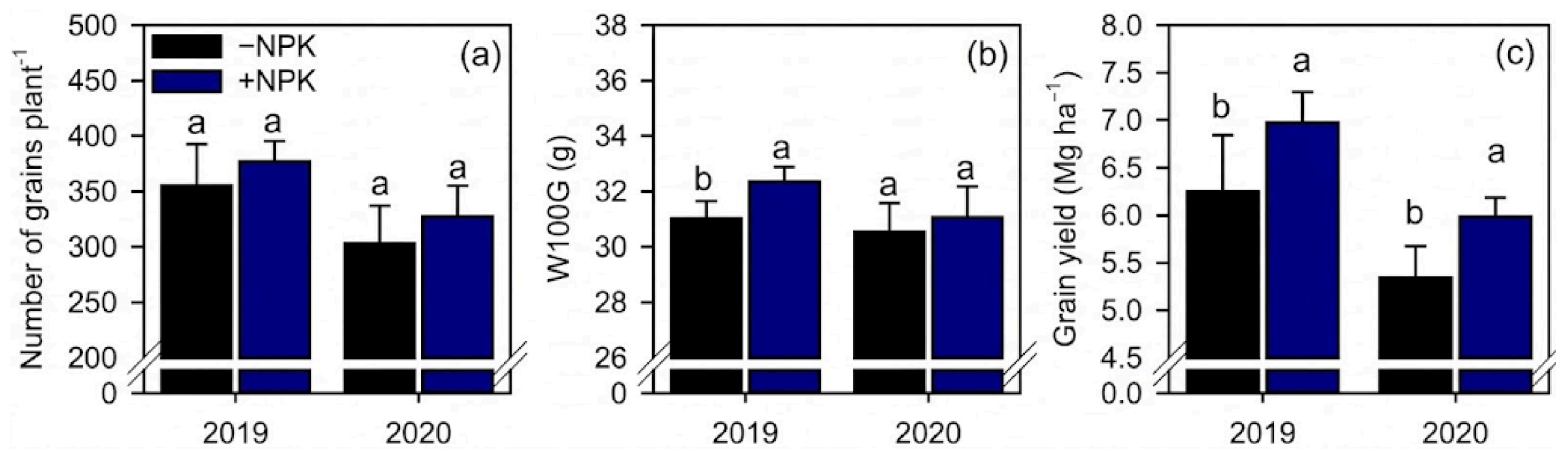
Off-season maize (**a**) number of grains per plant, (**b**) 100 grain weight (W100G), and (**c**) grain yield as a function of the presence or absence of foliar NPK application (+NPK or −NPK). Different letters indicate significant differences between treatments according to the F-test (*p* ≤ 0.10). Error bars indicate the standard deviation (*n* = 12).

Foliar NPK application generally improved maize yield components. Although prolificacy was not affected by NPK application, increases in the number of rows per ear and grains per row were observed (Tables S2 and S3). These responses were accompanied by numerical increases in the number of grains per plant of 5.9% in 2019 and 10.0% in 2020, although these differences were not statistically significant (Figure 8a). In contrast, 100-grain weight (W100G) increased significantly by 4.2% in 2019, whereas the 1.8% increase observed in 2020 was not significant (Figure 8b). Grain yield was significantly higher under +NPK in both growing seasons, exceeding −NPK by 0.72 Mg ha^−1^ in 2019 and 0.64 Mg ha^−1^ in 2020 (Figure 8c).

## 3. Discussion

Combined foliar application of nitrogen (N), phosphorus (P), and potassium (K), hereafter referred to as NPK, modulated the efficiency of the photosynthetic system in off-season maize under contrasting environmental conditions during crop development. Collectively, the increase ribulose-1,5-bisphosphate carboxylase/oxygenase (Rubisco) activity, optimization of gas exchange, and accumulation of primary metabolites indicate that foliar NPK application promoted metabolic adjustment and sustained carbon assimilation. These responses contributed to maintaining maize performance under off-season growing conditions.

In the present study, off-season maize establishment occurred under favorable water availability. However, as autumn progressed and winter began, the reduction in precipitation intensified the water deficit during critical stages for yield formation, including flowering and grain filling. Under these conditions, foliar NPK application prior to the intensification of environmental constraints improved off-season maize performance. These results are consistent with prior observations that foliar NPK supplementation can enhance plant metabolism and consequently increase grain yield [21,39,40,41].

Foliar NPK application increased leaf N and molybdenum (Mo) concentrations in both growing seasons and sulfur (S) concentrations in the second growing season. These nutritional responses may be associated with physiological relationships among nutrients involved in N metabolism. Nitrogen plays a central role in the synthesis of photosynthetic proteins, including Rubisco, as well as chlorophylls and cytochromes [42], although this mechanism was not directly evaluated in the present study. Molybdenum is an essential cofactor of nitrate reductase, whereas sulfur (S) is required for the synthesis of sulfur-containing amino acids and iron–sulfur proteins, including ferredoxin, which participates in photosynthetic electron transport [43,44]. In addition, P contributes to adenosine triphosphate (ATP)-dependent metabolic processes, while K is involved in enzyme activation and ionic homeostasis, both of which are important for plant metabolism [27]. These established physiological roles provide a plausible basis for interactions among nutrients following foliar NPK supplementation, but do not demonstrate a direct synergistic effect in the present experiment. Moreover, leaf N, P, and K concentrations remained within the adequate ranges reported for maize (27–35, 2.0–4.0, and 17–35 g kg^−1^, respectively) [45,46].

Following the increase in leaf nutrient concentrations of off-season maize, foliar NPK application enhanced photosynthetic performance during the 2020 growing season, as evidenced by increased net photosynthetic rate (*A*), water use efficiency (*WUE*), and apparent carboxylation efficiency (*A*/*Ci*), accompanied by reduced intercellular CO_2_ concentration (*Ci*). Interestingly, stomatal conductance (*gs*) and transpiration rate (*E*) were not significantly affected by foliar NPK application. The simultaneous increase in *A* and decrease in *Ci*, without significant changes in *gs*, suggests that the improvement in carbon assimilation may have been primarily associated with non-stomatal mechanisms, potentially involving enhanced biochemical capacity for CO_2_ fixation [47]. Furthermore, the increase in WUE was mainly associated with higher carbon assimilation rather than a reduction in transpirational water loss, indicating a more favorable balance between photosynthetic carbon gain and water expenditure under the field conditions evaluated.

In contrast to Rubisco, phosphoenolpyruvate carboxylase (PEPcase) activity was not significantly affected by foliar NPK application in either growing season. As PEPcase participates in the initial fixation of CO_2_ in C_4_ plants [48], its unchanged activity indicates that the improvements in photosynthetic performance observed in 2020 were not accompanied by increased activity of this enzyme. These findings suggest a differential response of carboxylation enzymes to foliar NPK supplementation, indicating that the effects on carbon assimilation were not associated with a uniform increase in the activities of both enzymes [49].

In parallel with the improvements in photosynthetic performance, foliar NPK application was associated with lower oxidative stress indicators in both growing seasons. Treated plants exhibited significantly lower hydrogen peroxide (H_2_O_2_) concentrations and malondialdehyde (MDA) levels than untreated plants, indicating reduced H_2_O_2_ accumulation and lipid peroxidation, respectively. Interestingly, these reductions were accompanied by lower activities of the antioxidant enzymes superoxide dismutase (SOD) and catalase (CAT), although the decrease in SOD activity was significant only in 2020. The simultaneous reduction in H_2_O_2_, MDA, and antioxidant enzyme activities suggests that foliar NPK application may have been associated with reduced oxidative pressure, thereby decreasing the demand for enzymatic antioxidant defense [50,51]. Since SOD catalyzes superoxide dismutation to H_2_O_2_ and CAT participates in H_2_O_2_ detoxification, their lower activities, together with reduced oxidative damage indicators, may reflect a lower requirement for reactive oxygen species scavenging rather than impaired antioxidant protection [52]. Moreover, the higher photosynthetic activity observed in 2020 may have contributed to a more favorable balance between carbon assimilation and cellular redox status.

Although foliar NPK application did not significantly increase leaf P and K concentrations, these nutrients may still contribute to physiological processes associated with carbon and nitrogen metabolism. Phosphorus plays a central role in ATP-mediated energy transfer, ribulose-1,5-bisphosphate (RuBP) regeneration, and the coordination of Rubisco activity with cellular energy availability [27,53,54]. Potassium, in turn, contributes to osmotic regulation, cellular ionic balance, and enzyme activation [55,56]. Furthermore, P supports N uptake and nitrate assimilation in maize [57,58], whereas K acts as a counterion during nitrate transport through the xylem and contributes to its redistribution via the phloem [59]. These established physiological functions provide a plausible basis for complementary effects of N, P, and K on plant metabolism. However, the present experimental design does not allow us to determine whether these interactions directly contributed to the physiological responses observed following foliar NPK application.

These results are consistent with the argument that foliar fertilization should not be considered solely as a nutritional input but also as a physiological strategy capable of enhancing metabolic activity and improving overall plant functioning [19]. According to [60,61,62], foliar nutrient application mitigates factors that impair physiological efficiency, promoting yield increases of approximately 15–19% and improvements in grain quality of 9–29% under different environmental stress conditions. Although foliar fertilization of maize plants can be a useful tool for supplementing nutrients, it should be used in conjunction with other fertilization methods and with careful attention to timing and application rates. The amount of nutrients that can be absorbed by leaves is limited, and most nutrient uptake by the plant occurs through the roots. Therefore, the primary aim of foliar fertilization should be to improve plant metabolism during periods of extreme environmental conditions.

The observed physiological responses were accompanied by increased leaf protein content in 2019 and Rubisco activity in 2020, whereas total soluble sugar concentrations increased significantly in both growing seasons. Rubisco accounts for about 50% of total protein, making it the most abundant protein in leaves [63]. These responses are consistent with the observed increases in leaf protein content and Rubisco activity, ultimately leading to higher total sugar accumulation [64]. This increase in the production of photoassimilates probably promoted an increase in grain productivity. Although the number of grains per plant did not differ significantly between treatments, small numerical increases in this component, combined with greater 100-grain weight (W100G), may have exerted a cumulative effect on grain production when extrapolated to the plant population per unit area. This combined contribution provides a plausible explanation for the significant increases in grain yield observed in both growing seasons, even when individual yield components showed limited or nonsignificant responses.

The present study demonstrates physiological and productive benefits of combined NPK application, with improvements maintained despite water deficits during the reproductive phase in both seasons. Recent studies have projected that rising temperatures and altered precipitation regimes may constrain future maize production [65,66]. Collectively, these findings provide a physiological basis for foliar NPK application as a strategy to sustain off-season maize performance under increasing climatic constraints.

## 4. Materials and Methods

### 4.1. Characterization of the Experimental Area

The field trials were conducted during the off-season in 2019 and 2020, from March to early August in both years, at the Experimental Lageado Farm of São Paulo State University, São Paulo State, Brazil (48°26′ W, 22°51′ S, and 786 m above sea level). The climate according to the Köppen–Geiger classification is Cwa, i.e., tropical, with a rainy season in the summer and a dry season in the winter [67]. The minimum and maximum daily average temperatures are 17.1 °C and 23.3 °C, respectively, and the average annual rainfall is 1360 mm [68].

During the field trial, climatological variables such as precipitation, maximum and minimum temperatures, and daily reference evapotranspiration (ETr) were collected to compute the hydroclimatic balance (Figure 1). The hydroclimatic balance was calculated using an electronic spreadsheet [69] following the procedures of [70]. The chemical and physical properties of the soil at a depth of 0–0.20 m were determined prior to the start of the field trials and are shown in Supplementary Table S1.

### 4.2. Experimental Design and Treatments

The treatments consisted of the presence (+NPK) and absence (−NPK) of foliar application of an NPK-based formulation and were arranged in randomized blocks with twelve replications. In both seasons, foliar applications were performed at maize phenological stage V_8_ using 4 L ha^−1^ of NPK formulation [852 g of N (urea), 248 g of P (phosphoric acid), 707 g of K (potassium chloride); Ubyfol, Uberaba, Brazil] with a vegetable oil adjuvant (30 mL ha^−1^) in a spray volume of 180 L ha^−1^. The product (NPK) dosage was chosen based on the manufacturer’s recommendation for the crop. For the control treatment, only water and adjuvant were applied to ensure identical application conditions across treatments, with the presence or absence of foliar NPK being the sole experimental difference. Spraying was conducted using a CO_2_-powered airbrush atomizer at a working pressure of 1.8 bar. The boom featured six flat fan nozzles (TTI 110 02 VP) spaced 0.5 m apart, operated at a velocity of 1 m s^−1^.

### 4.3. Maize Cultivation

Seeds of the maize hybrid P3707VYH (Pioneer®, Corteva Agriscience, Inc., Johnston, Iowa, USA) were treated with fungicides (carboxin + thiram, 1 g i.a. kg^−1^ of seed) before sowing. The experimental plots consisted of ten 10 m-long rows spaced 0.45 m apart. The sowing density was 3.1 seeds per m^−1^ to obtain approximately 68.888 plants per ha^−1^. At sowing in both seasons, fertilization was performed with 300 kg ha^−1^ of the 28-08-16 formula (24 kg ha^−1^ of N; 37.5 kg ha^−1^ of P, and 39.8 kg ha^−1^ of K). Top dressing fertilization was carried out at the V_6_ phenological stage with the application of 100 kg ha^−1^ of N in the form of ammonium sulfate and 16.6 kg ha^−1^ of K in the form of potassium chloride [45]. Pest, disease, and weed management were conducted as recommended for maize [71].

### 4.4. Assessments and Analysis

#### 4.4.1. Sampling Procedures and Timing of Assessments

Foliar NPK application was performed at the V_8_ phenological stage, and physiological and biochemical assessments were conducted at the R1 stage (flowering), approximately 20 days after application. For nutritional analysis, 20 ear leaves per plot were collected, using the middle third of each leaf. Gas exchange measurements were performed in 10 fully expanded leaves per plot, whereas biochemical analyses were conducted using 20 leaves collected from the leaf immediately below and opposite the primary ear of maize plants. Leaf samples for biochemical determinations were pooled within each plot, immediately frozen in liquid nitrogen, and stored at −80 °C until analysis. Samples were subsequently processed under controlled laboratory conditions for the determination of enzyme activities, protein concentration, soluble sugars, and oxidative stress indicators. All measurements were performed using the same sampling procedures across treatments and growing seasons.

#### 4.4.2. Nutritional Analysis

To determine the nutritional status of the maize plants, leaf samples were collected at the R_1_ stage from the middle third of 20 ear leaves per plot. The N concentration was determined using the semi-micro Kjeldahl method, while the concentrations of P, K, calcium (Ca), magnesium (Mg), sulfur (S), boron (B), iron (Fe), molybdenum (Mo), copper (Cu), manganese (Mn), and zinc (Zn) were obtained after nitric–perchloric digestion and quantified according to the procedures described by [46].

#### 4.4.3. Gas Exchange

Gas exchange measurements were performed exclusively during the 2020 growing season, at the R_1_ phenological stage, as this assessment was incorporated into the physiological evaluation protocol in the second year of the experiment. Photosynthetic parameters were determined using a model Ciras-3 portable gas exchange device (Portable Photosynthesis System, PP Systems Inc., Amesbury, MA, USA). The instrument measurement conditions were standardized to 380–400 mol^−1^ atmospheric CO_2_, controlled artificial illumination provided by LED lamps at a constant photosynthetic photon flux density (PPFD) of 1100 µmol photons m^−2^ s^−1^. The average leaf chamber temperature recorded during the measurements ranged from 25 to 27 °C. Measurements were conducted between 10:00 a.m. and 12:00 p.m. The analyzed parameters included the net photosynthetic rate (*A*, µmol CO_2_ m^−2^ s^−1^), stomatal conductance (*gs*, mol H_2_O m^−2^ s^−1^), internal CO_2_ concentration (*Ci*, µmol mol), transpiration rate (*E*, mmol H_2_O m^−2^ s^−1^), water use efficiency [*WUE*, µmol CO_2_ (mmol H_2_O) ^−1^] determined from *A*/*E*, and carboxylation efficiency determined from *A*/*Ci*.

#### 4.4.4. Carboxylation Enzymes Activity

The supernatant used to determine enzyme activity and protein concentration was obtained by grinding fresh leaves with a mortar and pestle pre-cooled with liquid nitrogen. To this sample, buffer containing 50 mM Tris-HCl (pH 7.8), 2 mM Na_2_EDTA, 5 mM β-mercaptoethanol (βME), 1 mM PMSF, and 10 kg m^−3^ PVP was added. The resulting homogenate was centrifuged at 1500× *g* for 30 min at 4 °C [72].

Phosphoenolpyruvate carboxylase (PEPcase) and Ribulose-1,5-bisfosfato carboxilase/oxygenase (Rubisco) activities were determined using the same leaf extract. PEPcase (EC 4.1.1.31) activity was measured spectrophotometrically by monitoring NADH oxidation at 340 nm through a reaction coupled to malate dehydrogenase and initiated by the addition of phosphoenolpyruvate (PEP). The decrease in absorbance was recorded over 300 s at 25 °C [72]. Rubisco (EC 4.1.1.39) activity was determined spectrophotometrically at 340 nm using a separate coupled enzymatic reaction initiated by the addition of ribulose-1,5-bisphosphate (RuBP). Rubisco activity was calculated from the difference in absorbance readings between 0 and 1 min [73]. Both enzyme activities were expressed in μmol min^−1^ mg^−1^ protein.

#### 4.4.5. Oxidative Stress Markers and Antioxidant Enzymes Activity

Lipid peroxidation was assessed by determining malondialdehyde (MDA) concentration using the thiobarbituric acid (TBA) reaction method, with absorbance measured at 532 nm and corrected at 600 nm. Results were expressed as nmol g^−1^ fresh weight (FW) [74]. Hydrogen peroxide (H_2_O_2_) concentration was determined following extraction with 0.1% trichloroacetic acid (TCA) and reaction with potassium iodide (KI), with absorbance measured at 390 nm and concentrations calculated using an H_2_O_2_ standard curve [75]. Superoxide dismutase (SOD; EC 1.15.1.1) activity was determined based on the inhibition of nitroblue tetrazolium (NBT) photoreduction at 560 nm, with results expressed as U mg^−1^ protein [76]. Catalase (CAT; EC 1.11.1.6) activity was evaluated by monitoring H_2_O_2_ decomposition through the decrease in absorbance at 240 nm, with results expressed as µmol min^−1^ mg^−1^ protein [77].

#### 4.4.6. Total Concentration of Soluble Sugar

Total leaf total protein concentrations were determined according to the method proposed by [78] using a standard curve constructed from bovine serum albumin, with results expressed in mg g^−1^ of fresh weight (FW).

The total concentration of soluble sugars in maize leaves was determined using the phenol–sulfuric acid method, in which carbohydrates react with phenol under strongly acidic conditions to produce a colored complex. Absorbance was measured spectrophotometrically at 490 nm. Concentrations were calculated using a sucrose standard curve and expressed as g sucrose equivalents kg^−1^. Concentrations were determined using a standard curve of sucrose, with results expressed in g kg^−1^ DW [79].

#### 4.4.7. Agronomic Parameters and Grain Yield

When the maize plants reached physiological maturity, a 15-m^2^ area from each plot was harvested, and the following variables were evaluated: prolificacy (number of ears per plant), number of grains per ear, 100 grain weight (P100G) expressed in g, and grain yield expressed in Mg ha^−1^, with its moisture content adjusted to 13% using an automatic moisture meter (Gehaka G650i, Ind. e Com. Eletrônica Gehaka Ltda., São Paulo, SP, Brazil).

### 4.5. Statistical Analysis

The data were assessed for normality [80] and homoscedasticity [81], considering a significance threshold of *p* ≤ 0.10. Individual analyses were conducted for each growing season, followed by a combined analysis using data from both growing seasons (Table S2). For the combined analysis, a linear mixed-effects model was specified, with foliar NPK application considered a fixed effect, while growing season, the growing season × NPK interaction, and blocks nested within growing seasons were treated as random effects. This model accounted for variability among growing seasons and experimental blocks, allowing treatment effects to be evaluated using appropriate error terms. The significance threshold of *p* ≤ 0.10 was adopted to increase sensitivity to physiological and biochemical responses under variable field conditions, following the approach used in previous agronomic studies [20,44,82]. This criterion was applied across the statistical analyses, recognizing the increased probability of Type I errors compared with the conventional 5% threshold. Exact *p*-values were reported to distinguish significant effects at *p* ≤ 0.05 from those significant only at 0.05 < *p* ≤ 0.10. Statistical analyses were performed using R software (version 2026.05.0+218).

## 5. Conclusions

In this study, foliar application of NPK was associated with increases in Rubisco activity, the net photosynthetic rate, stomatal conductance, water use efficiency, carboxylation efficiency, and total protein and sugar concentrations in off-season maize. These responses included increased Rubisco activity, net photosynthetic rate, water use efficiency, apparent carboxylation efficiency, and leaf protein and soluble sugar concentrations, along with reduced oxidative stress, as evidenced by lower H_2_O_2_ accumulation and lipid peroxidation (MDA). The concomitant reduction in antioxidant enzyme activities suggests a lower demand for enzymatic ROS scavenging, consistent with improved cellular oxidative balance. Together, these physiological and biochemical responses were associated with improvements in grain yield under off-season growing conditions. The findings reinforce the relevance of foliar fertilization as a complementary management practice to improve off-season maize performance. Future studies could optimize the effectiveness of foliar NPK application by evaluating different rates and phenological stages and include individual and combined applications of N, P, and K to distinguish their respective contributions and identify potential additive or synergistic effects on maize physiological performance and grain yield.

## Data Availability

The datasets analyzed during the current study are available from the corresponding author upon reasonable request.

## Supplementary Materials

The following supporting information can be downloaded at: https://www.mdpi.com/article/10.3390/plants15213484/s1, Table S1. Initial soil physicochemical characteristics (0–0.20 m depth) before sowing. Table S2. Summary of the combined analysis of variance (ANOVA) for nutrient concentration, carbon assimilation, protein and total soluble sugar contents, agronomic parameters, and grain yield in maize as affected by growing season and the presence or absence of foliar NPK application. Table S3. *p*-values for the effects of foliar NPK application on leaf nutrient concentrations, gas exchange, photosynthetic enzyme activities, oxidative stress indicators, antioxidant enzyme activities, protein and soluble sugar concentrations, yield components, and grain yield of off-season maize during the 2019 and 2020 growing seasons.
