# Supplementary material for: Foliar Supplementation with NPK Stimulates Carbon Metabolism and Improves Water Use Efficiency and Off-Season Maize Crop Yield
Source: Plants (Basel). 2026 Nov 8;15(21):3484. doi: 10.3390/plants15213484 (PMC13766109; doi:10.3390/plants15213484)
Supplement: Supplementary file 1 [file plants-15-03484-s001.zip › plants-4534206-supplementary.pdf]

Table S1. Initial soil physicochemical characteristics (0–0.20 m depth) before sowing.

**Physical properties**			
**Clay**	**Silt**	**Sand**	**Bulk density**
g kg^{-1}	g kg^{-1}	g kg^{-1}	g cm^{-3}
612	271	117	1.23

**Soil fertility**									
**pH ($CaCl_2$)**	**Organic matter**	**Available P**	**K^+**	**Ca^{2+}**	**Mg^{2+}**	**Al^{3+}**	**Potential acidity (H + Al)**	**Base saturation**	**CEC (pH 7.0)**
—	g kg^{-1}	mg kg^{-1}	mmolc kg^{-1}	mmolc kg^{-1}	mmolc kg^{-1}	mmolc kg^{-1}	mmolc kg^{-1}	%	mmolc kg^{-1}
5.15	27.4	49	3.9	25	15	0.31	40	51	86

**Sulfur and micronutrients**					
**S–SO_4^{2-}**	**B**	**Cu**	**Fe**	**Mn**	**Zn**
mg kg^{-1}	mg kg^{-1}	mg kg^{-1}	mg kg^{-1}	mg kg^{-1}	mg kg^{-1}
4.72	0.4	8.8	22	21.2	2.1

Notes. CEC, cation exchange capacity; DTPA-TEA, diethylenetriaminepentaacetic acid–triethanolamine. Available phosphorus (P) and exchangeable potassium (K), calcium (Ca), and magnesium (Mg) were determined using resin extraction; aluminum (Al) using KCl; sulfur (S–SO_4^{2-}) using calcium phosphate; boron (B) using hot water; and copper (Cu), iron (Fe), manganese (Mn), and zinc (Zn) using DTPA-TEA. Source attribution should be added if data are reproduced or adapted from another publication.

.

Table S2. Summary of the combined analysis of variance (ANOVA) for nutrient concentration, carbon assimilation, protein and total soluble sugar contents, agronomic parameters, and grain yield in maize as affected by growing season and the presence or absence of foliar NPK application.

	Nutrient concentrations										
	**N**	**P**	**Ca**	**Mg**	**S**	**Fe**	**Cu**	**Zn**	**Mn**	**Mo**	**B**
**Anova (F probability)**											
**Growing season (GS)**	0,3192	< 0,0001	1,0000	0,0002	< 0,0001	0,8597	0,2219	0,0966	< 0,0001	0,0022	0,8073
**Foliar NPK (NPK)**	<0,0001	0,0051	0,2790	< 0,0001	< 0,0001	0,2080	0,1680	0,1080	0,3850	< 0,0001	0,3258
**GS × NPK**	0,8657	0,3650	0,4536	0,8388	0,2391	0,0568	0,4086	0,5372	0,7217	0,1632	0,8659
**C.V. (%)**	4,7102	5,0714	17,0484	7,1922	9,5292	5,8724	10,1426	7,3909	6,2200	9,3415	12,1162

	Carbon assimilation, protein and total soluble sugar content			
	**PEPcase**	**Rubisco**	**Leaf protein**	**Total sugar**
**Anova (F probability)**				
**Growing season (GS)**	< 0.0001	0.9834	0.2796	0.0011
**Foliar NPK (NPK)**	0.1299	< 0.0001	0.0013	< 0.0001
**GS × NPK**	0.2177	0.9173	0.8701	0.7112
**C.V. (%)**	10.5530	11.5871	11.5498	5.8614

	Agronomic parameters and grain yield			
	**Grains per ear**	**100-grain weight (W100G)**	**Grain yield**	**Prolificacy (ears plant^{-1})**
**Anova (F probability)**				
**Growing season (GS)**	< 0.0001	0.0010	< 0.0001	0.6843
**Foliar NPK (NPK)**	< 0.0001	0.0006	< 0.0001	0.5586
**GS × NPK**	0.2531	0.1233	0.6739	0.8159
**C.V. (%)**	4.5991	2.7436	5.4841	4.7700

Nitrogen (N); phosphorus (P); calcium (Ca); magnesium (Mg); sulfur (S); iron (Fe); copper (Cu); zinc (Zn); manganese (Mn); molybdenum (Mo); boron (B); phosphoenolpyruvate carboxylase (PEPcase); ribulose-1,5-bisphosphate carboxylase/oxygenase (Rubisco); leaf protein concentration; total soluble sugars; number of grains per ear; 100-grain weight (W100G); grain yield; ear prolificacy (ears plant^{-1}).

GS = growing season; NPK = presence or absence of foliar NPK application; GS × NPK = interaction between growing season and foliar NPK application. Significant effects were considered at $p \leq 0.10$.

Table S3. *p*-values for the effects of foliar NPK application on leaf nutrient concentrations, gas exchange, photosynthetic enzyme activities, oxidative stress indicators, antioxidant enzyme activities, protein and soluble sugar concentrations, yield components, and grain yield of off-season maize during the 2019 and 2020 growing seasons.

Variable	2019 p value	2020 p value
**Leaf macronutrients**		
Nitrogen (N)	0.026	0.100
Phosphorus (P)	0.292	0.308
Potassium (K)	0.525	0.138
Calcium (Ca)	0.263	0.249
Magnesium (Mg)	0.094	0.185
Sulfur (S)	0.100	0.017
**Leaf micronutrients**		
Boron (B)	0.756	0.784
Iron (Fe)	0.130	0.871
Molybdenum (Mo)	0.029	0.008
Copper (Cu)	0.226	0.432
Manganese (Mn)	0.486	0.505
Zinc (Zn)	0.468	0.303
**Gas exchange**		
Net photosynthetic rate (A)	-	0.0066
Stomatal conductance (gs)	-	0.1754
Intercellular CO_2 concentration (Ci)	-	0.008

Transpiration (E)	-	0.2169
Water use efficiency (WUE)	-	0.0463
Carboxylation efficiency (A/Ci)	-	0.002
**Photosynthetic enzymes**		
PEP carboxylase (PEPcase)	0.698	0.466
Rubisco	0.248	0.025
**Oxidative stress and antioxidant enzymes**		
Hydrogen peroxide (H_2O_2)	0.023	0.015
Malondialdehyde (MDA)	0.050	0.014
Superoxide dismutase (SOD)	0.235	0.036
Catalase (CAT)	0.036	<0.001
**Protein and soluble sugars**		
Leaf protein	0.096	0.400
Total soluble sugars	0.001	0.003
**Yield components and grain yield**		
Grains per plant	0.420	0.211
100-grain weight (W100G)	0.009	0.590
Grain yield	0.048	0.091
Prolificacy (ears plant^{-1})	0.6376	0.4765
